# Serum interferon-λ3 as a short-term biomarker of disease control in anti-MDA5-positive dermatomyositis-associated ILD

**DOI:** 10.1038/s41598-026-37104-x

**Published:** 2026-01-24

**Authors:** Yoshihiro Kitahara, Tomoyuki Fujisawa, Atsuki Fukada, Keigo Koda, Taisuke Akamatsu, Masaki Ikeda, Masato Fujii, Mitsuru Niwa, Yusuke Kaida, Hiroyuki Matsuda, Koshi Yokomura, Naoki Koshimizu, Mikio Toyoshima, Shiro Imokawa, Dai Hashimoto, Keita Yamashita, Moriya Iwaizumi, Masato Maekawa, Yusuke Inoue, Hideki Yasui, Hironao Hozumi, Yuzo Suzuki, Masato Karayama, Kazuki Furuhashi, Noriyuki Enomoto, Naoki Inui, Takafumi Suda

**Affiliations:** 1 Second Division, Department of Internal Medicine, Hamamatsu University School of Medicine, 1-20-1 Handayama Chuo-ku, Hamamatsu, Shizuoka 431-3192 Japan; 2 Department of Respiratory Medicine, Hamamatsu Rosai Hospital, Hamamatsu, Japan; 3 Department of Respiratory Medicine, Shizuoka General Hospital, Shizuoka, Japan; 4 Department of Respiratory Medicine, Shizuoka Saiseikai General Hospital, Shizuoka, Japan; 5 Department of Respiratory Medicine, Shizuoka City Shizuoka Hospital, Shizuoka, Japan; 6 Department of Respiratory Medicine, Hamamatsu Medical Center, Hamamatsu, Japan; 7 Department of Respiratory Medicine, Enshu Hospital, Hamamatsu, Japan; 8 Department of Respiratory Medicine, Japanese Red Cross Shizuoka Hospital, Shizuoka, Japan; 9 Department of Respiratory Medicine, Seirei Mikatahara General Hospital, Hamamatsu, Japan; 10 Department of Respiratory Medicine, Fujieda Municipal General Hospital, Fujieda, Japan; 11 Department of Respiratory Medicine, Iwata City Hospital, Iwata, Japan; 12 Department of Respiratory Medicine, Seirei Hamamatsu General Hospital, Hamamatsu, Japan; 13 Department of Laboratory Medicine, Hamamatsu University School of Medicine, Hamamatsu, Japan; 14 Department of Clinical Pharmacology and Therapeutics, Hamamatsu University School of Medicine, Hamamatsu, Japan

**Keywords:** Interferon-lambda 3, Polymyositis, Dermatomyositis, Interstitial lung disease, Melanoma differentiation-associated gene 5, Biomarkers, Diseases, Immunology, Medical research

## Abstract

This study aimed to assess the clinical utility of serum interferon-lambda 3 (IFN-λ3) as a sequential biomarker for treatment response and disease control in patients with anti-melanoma differentiation-associated gene 5 (MDA5) antibody-positive dermatomyositis (DM)-associated interstitial lung disease (ILD). Serum IFN-λ3 levels were measured in 24 patients with anti-MDA5 antibody-positive DM-ILD at diagnosis and 1 month after initiating immunosuppressive therapy. Patients were categorized into two groups based on clinical outcomes: a good control group (*n* = 16; survived without relapse for ≥ 1 year) and a poor control group (*n* = 8; died from ILD progression or relapse within 1 year). Changes in serum IFN-λ3 levels and differences between groups were analyzed. In the good control group, serum IFN-λ3 levels significantly decreased from 94.6 to 12.7 pg/mL (*p* < 0.001), whereas no significant change was observed in the poor control group (129.0 to 118.8 pg/mL). Furthermore, serum IFN-λ3 levels at 1 month were significantly lower in the good control group than in the poor control group (*p* = 0.004). Serum IFN-λ3 levels may reflect short-term treatment response and could serve as a useful sequential biomarker for assessing disease control in patients with anti-MDA5 antibody-positive DM-ILD.

**Supplementary Information:**

The online version contains supplementary material available at 10.1038/s41598-026-37104-x.

## Introduction

Idiopathic inflammatory myopathies (IIMs) are a group of autoimmune diseases characterized by muscle involvement and extra-muscular manifestations, including those affecting the skin and lungs. The lungs are one of the most commonly affected extramuscular organs in IIM, and interstitial lung disease (ILD) is a prevalent and often devastating complication. Recent studies have demonstrated that the presence of myositis-specific antibodies, such as anti-melanoma differentiation-associated gene 5 (MDA5) and anti-aminoacyl-tRNA synthetase (ARS) antibodies, is correlated with clinical features, the frequency and severity of organ involvement and prognosis in patients with IIM. ILD is particularly common in patients with antisynthetase syndrome and anti-MDA5 antibody-positive dermatomyositis (DM).

The presence of anti-MDA5 antibodies is recognized as a poor prognostic marker in patients with IIM-associated ILD^1–3^. Anti-MDA5 antibodies are detected in 30%–50% of patients with DM-associated ILD (DM-ILD)^1,2,4^ and are strongly associated with the development of rapidly progressive ILD, which can lead to fatal outcomes. Previous studies have identified several prognostic markers in patients with anti-MDA5 antibody-positive DM-ILD, including elevated serum Krebs von den lungen-6 (KL-6)^5^ and ferritin levels^6,7^. However, accurately assessing disease severity and predicting initial treatment responses remains a clinical challenge. In particular, sensitive biomarkers that reflect disease control and treatment response within the short term (e.g. within 1 month of treatment initiation) are currently lacking in routine clinical practice for this patient population.

Interferon-lambda (IFN-λ), a type III interferon, exerts antiviral activity and regulates immune responses at epithelial barrier surfaces^8,9^. The IFN-λ family includes four members: IFN-λ1, IFN-λ2, IFN-λ3 and IFN-λ4^10^. Recent studies have implicated activation of the IFN-λ pathway in the pathogenesis of autoimmune diseases, such as systemic lupus erythematosus (SLE), systemic sclerosis (SSc) and IIM^11–13^. In the context of IIM-associated ILD, we previously reported that serum IFN-λ3 levels were selectively elevated in patients with anti-MDA5 antibody-positive DM-ILD among those with polymyositis/dermatomyositis-associated ILD (PM/DM-ILD) and that high serum IFN-λ3 levels at the time of diagnosis were significantly associated with poor prognosis in patients with anti-MDA5 antibody-positive DM-ILD^14^. Furthermore, Yoshida et al. reported that anti-MDA5 antibody-positive DM patients with marked elevation of serum IFN-λ3 levels tended to present with rapidly progressive ILD and were associated with poor prognosis^15^. These findings suggest that serum IFN-λ3 may serve as a prognostic marker in patients with anti-MDA5 antibody-positive DM-ILD. However, it remains unclear whether serum IFN-λ3 levels reflect short-term treatment response and disease control during the clinical course, and how these levels change over time in response to therapy.

This study aimed to investigate short-term changes in serum IFN-λ3 levels and to evaluate the clinical utility of IFN-λ3 as a sequential biomarker of treatment response and disease control in patients with anti-MDA5 antibody-positive DM-ILD.

## Methods

### Study design and participants

This retrospective study included patients with anti-MDA5 antibody-positive DM-ILD diagnosed at Hamamatsu University Hospital and its affiliated hospitals between January 1996 and December 2022 and for whom stored serum samples were available at diagnosis and 1 month after treatment initiation. Data analyses were conducted from December 2023 to March 2024. The observation period extended from the date of DM-ILD diagnosis to the last follow-up visit (censoring or death).

The diagnosis of DM was based on the Bohan and Peter criteria or the 2017 EULAR/ACR classification criteria^16,17^. Elevated anti-MDA5 antibody titers were used as reference findings. Clinically amyopathic dermatomyositis (CADM) was diagnosed based on the presence of characteristic skin rashes (e.g. Gottron’s papules, heliotrope rash, skin ulcers, mechanic’s hands) in the absence of muscular symptoms and with minimal or no elevation in serum creatine kinase levels.

ILD was classified into three forms: acute, subacute and chronic. Acute ILD was defined as a rapidly progressive form, characterized by acute worsening or new-onset dyspnea with widespread alveolar abnormalities on chest high-resolution computed tomography (HRCT) occurring within 1 month from the onset of respiratory symptoms or the first clinical visit^18–20^. Subacute ILD was defined as ILD with progressive respiratory deterioration occurring within 1–3 months^18–20^. Chronic ILD was defined as stable or slowly progressive ILD, presenting with gradual deterioration over a period longer than 3 months^18–20^.

The study protocol was approved by the Ethics Committee of Hamamatsu University School of Medicine (Approval Number: 18-030). The requirement for informed consent was waived due to the retrospective nature of the study. All procedures were conducted in accordance with the approved guidelines.

### Measurement and evaluation of serum biomarkers

Serum IFN-λ3 levels were measured using stored serum samples obtained during routine clinical practice at the time of diagnosis and 1 month after treatment initiation in patients with anti-MDA5 antibody-positive DM-ILD. Quantification of serum IFN-λ3 was performed using a SYSMEX IFN-λ3 assay kit (HISCL™ IFN-λ3 reagent; Sysmex Corp., Kobe, Japan), as previously described^21,22^. Briefly, serum IFN-λ3 levels were measured using a chemiluminescent enzyme immunoassay (CLEIA). The measurement range of the assay was 3.0–200.0 pg/mL. Values below the lower limit of quantification were treated as 3.0 pg/mL for statistical analyses, and samples exceeding the upper limit were re-measured after appropriate dilution according to the manufacturer’s instructions.

Anti-MDA5 antibodies were measured using a commercially available enzyme-linked immunosorbent assay (ELISA), and all samples were tested uniformly using the same assay method^23^. Serum KL-6, surfactant protein-D (SP-D), and ferritin levels were measured at either a central commercial laboratory (SRL Inc., Japan) or at the respective participating institutions as part of routine clinical practice. Depending on the measurement period and the institution, different immunoassay methods were used, including CLEIA and latex agglutination immunoassays, both of which are widely used and clinically validated in Japan.

### Classification of study participants

Patients were classified into two groups based on disease control of ILD following initial treatment: the good control group and the poor control group. Good control was defined as patients who responded to initial treatment and remained in stable condition without any ILD relapse for at least 1 year after initial treatment. Poor control was defined as patients who either died due to ILD progression despite treatment or experienced relapse of ILD within 1 year of initial treatment. Relapse of ILD was defined as the presence of at least two of the following criteria, based on the international consensus statement for idiopathic pulmonary fibrosis with slight modifications^24,25^: (1) Symptomatic exacerbation (e.g. dyspnea on exertion); (2) Increased opacities on chest X-ray or HRCT; and (3) At least one of the following physiological changes: > 10 mmHg decrease in arterial oxygen tension (PaO_2_), > 4% decrease in peripheral oxygen saturation (SpO_2_) or > 10% decrease in percent predicted forced vital capacity (%FVC; percent predicted vital capacity [%VC]).

### Data collection

Clinical data, including patient characteristics, symptoms, physical findings, treatment details, laboratory results and pulmonary function test data, were collected from electronic medical records. Muscle and skin manifestations specific to dermatomyositis were clinically evaluated at baseline, at 1 month after treatment initiation, and during follow-up, as deemed appropriate by the treating physicians.

### Statistical analysis

Categorical variables were expressed as numbers and percentage, while continuous variables were presented as medians with interquartile ranges (IQRs). The Mann–Whitney U test was used to compare continuous variables, and Fisher’s exact test was used for categorical variables. All statistical analyses were performed using EZR software version 1.55 (Saitama Medical Center, Jichi Medical University, Saitama, Japan)^26^. A *p* value of < 0.05 was considered statistically significant.

## Results

### Clinical characteristics of patients with anti-MDA5 antibody-positive DM-ILD

**Fig. 1:**
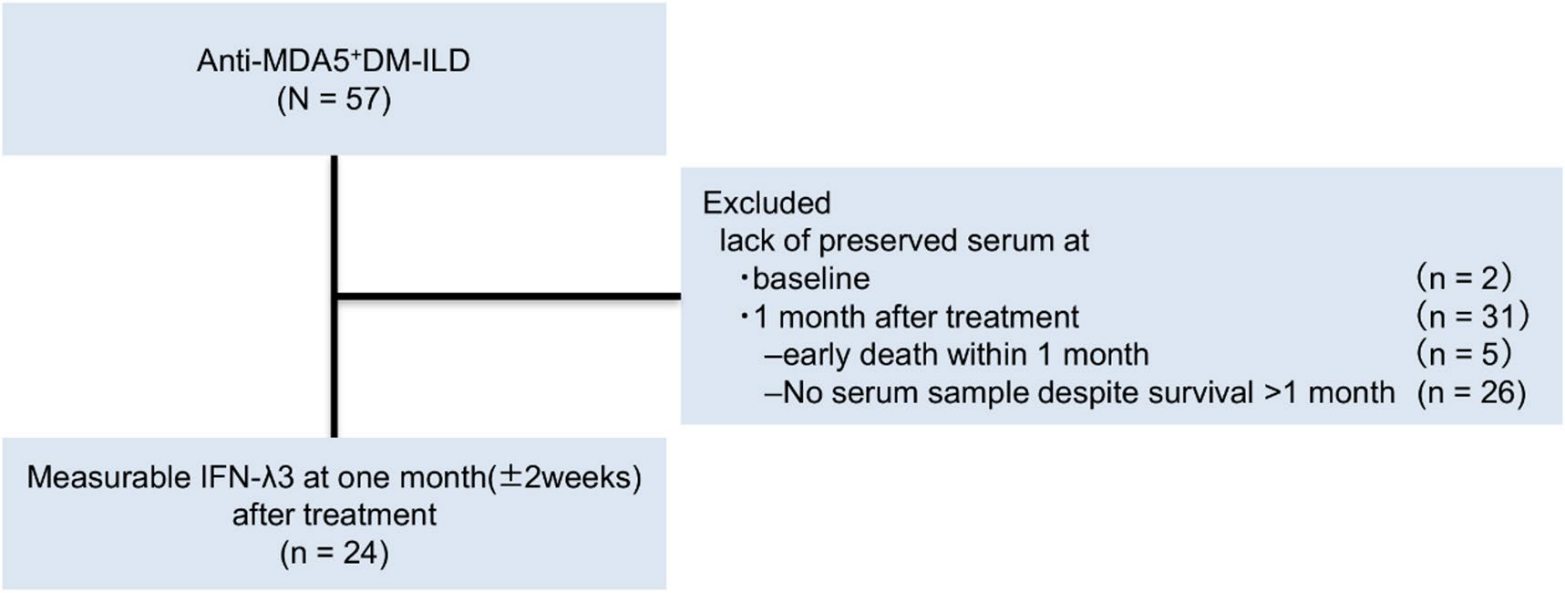
Study flow diagram.

A flowchart of patient selection for the study is presented in Fig. 1.

After screening and exclusion, 24 patients with anti-MDA5 antibody-positive DM-ILD who had stored serum samples at the time of diagnosis and 1 month after treatment initiation were included in this study.

DM, dermatomyositis; IFN-λ3, interferon-lambda 3; ILD, interstitial lung disease; MDA5, melanoma differentiation-associated gene 5.

Among 57 patients with anti-MDA5 antibody-positive DM-ILD, two were excluded due to the absence of stored serum samples at diagnosis and 31 were excluded due to a lack of stored samples at 1 month after treatment initiation (including 5 patients who died within 1 month). Finally, 24 patients with stored serum samples at both time points were included in this study.

The clinical characteristics of these 24 patients are summarized in Table 1.

**Table 1:** Clinical characteristics of the 24 patients with anti-MDA5 antibody-positive DM-ILD.

	All patients
	(*n* = 24)
Age, years	53 [47–65]
Sex, male	7 (29.2)
Diagnosis	
DM	9 (37.5)
CADM	15 (62.5)
Form of ILD	
Acute	7 (29.2)
Subacute	10 (41.7)
Chronic	7 (29.2)
S moking status, former	11 (45.8)
Symptoms	
Fever	10 (41.6)
Cough	14 (58.3)
Dyspnea	16 (66.7)
Fine crackles	21 (87.5)
Arthralgia	12 (50.0)
Gottron’s papule	21 (87.5)
Heliotrope rash	9 (37.5)
Skin ulcer	4 (16.7)
Muscle weakness	9 (37.5)
Muscle pain	8 (33.3)
Pulmonary function indices (*n* = 22)	
FVC, L	2.2 [1.7–2.7]
%FVC	75.7 [68.0–80.5]
FEV_1_, L	1.7 [1.5–2.7]
%FEV_1_	68.4 [57.0–84.0]
Pulmonary diffusion capacity (*n* = 17)	
DLco, mL/min/mmHg	13.2 [10.5–15.5]
%DLco	63.1 [54.6–85.3]
PaO_2_, Torr (*n* = 20)	77.5 [64.8–84.5]
P/F ratio (*n* = 23)	357 [292–401]
Treatment	
mPSL pulse therapy before maintenance therapy	13 (54.2)
CS + CNI	17 (70.8)
CS + CNI + cyclophosphamide	7 (29.2)
Mortality	6 (25.0)
Relapse of ILD < 1 year	2 (8.3)

Data are presented as the median [interquartile range] or number (%).

CADM, Clinically amyopathic dermatomyositis; CNI, Calcineurin inhibitor; DLco, Diffusion capacity of the lung for carbon monoxide; DM, Dermatomyositis; FEV_1_, Forced expiratory volume in 1 s; FVC, Forced vital capacity; ILD, Interstitial lung disease; MDA5, melanoma differentiation-associated gene 5; mPSL, Methylprednisolone; PaO_2_, Partial pressure of arterial oxygen; P/F ratio, PaO_2_/fraction of inspired oxygen ratio.

The median age was 53 years and 17 patients (70.8%) were women. None of the patients was positive for anti-ARS antibodies, whereas other myositis-specific or myositis-associated antibodies, including anti-Ro52 antibodies, were not systematically evaluated in this cohort. Regarding treatment, 17 patients (70.8%) received combination therapy with oral corticosteroids and calcineurin inhibitors, while 7 patients (29.2%) received triple therapy with corticosteroids, tacrolimus and intravenous cyclophosphamide. Despite intensive immunosuppressive therapy, six patients (25.0%) died of respiratory failure due to ILD progression within 1 year, without achieving meaningful clinical improvement after initial induction therapy. Among the 18 surviving patients, two experienced ILD relapse within the first year following initial treatment after a transient clinical response. Thus, eight patients (33.3%) were classified into the poor control group (six deaths and two relapses, which were considered insufficient control of ILD after initial treatment), and 16 patients were classified into the good control group. Detailed clinical characteristics of these two relapse cases are summarized in Supplementary Table S1. To assess the potential impact of selection bias, baseline clinical characteristics were compared between the 24 patients included in the present study and 31 patients excluded due to the lack of stored serum samples at 1 month. As shown in Supplementary Table S2, no significant differences were observed between the two groups in baseline clinical findings, serum IFN-λ3 levels, mortality, or relapse rates.

A comparison of clinical characteristics between the good control group and the poor control group is summarized in Table 2.

**Table 2:** Comparison of clinical characteristics between the good control group and the poor control group.

	Good control group	Poor control group	*p* value
	(*n* = 16)	(*n* = 8)	
Age, years	51 [43–61]	55 [52–67]	0.26
Sex, male	5 (31.2)	2 (25.0)	1
Diagnosis			
DM	6 (37.5)	3 (37.5)	0.66
CADM	10 (62.5)	5 (62.5)	
Form of ILD			
Acute	2 (12.5)	5 (62.5)	0.06
Subacute	8 (50.0)	2 (25.0)	
Chronic	6 (37.5)	1 (12.5)	
Smoking status, former	6 (37.5)	5 (62.5)	0.44
Pulmonary function indices (*n* = 22)			
FVC, L	2.3 [2.0–2.9]	1.7 [1.5–2.1]	0.07
%FVC	76.5 [68.2–82.3]	71.6 [46.2–86.3]	0.27
FEV_1_, L	1.7 [1.6–2.4]	1.6 [1.5–1.7]	0.15
%FEV_1_	73.0 [58.8–85.4]	59.8 [56.2–68.1]	0.34
Laboratory indices (at diagnosis)			
WBC, /μL	4900 [4185–5480]	4600 [4125–7125]	0.99
CRP, mg/dL	0.3 [0.2–1.6]	1.1 [0.7–1.6]	0.25
CK, U/L	55.5 [40.3–120.0]	181.0 [87.5–275.8]	0.20
Aldolase, U/L	8.1 [6.4–10.3]	7.4 [5.9–10.8]	0.83
Ferritin, ng/mL	407 [200–571]	862 [400–980]	0.14
KL-6, U/mL	742 [622–1031]	838 [547–1166]	0.95
SP-D, ng/mL	84.9 [40.7–108.6]	36.2 [27.8–51.5]	0.11
IFN-λ3, pg/mL	94.6 [21.1–120.8]	129.0 [74.6–231.0]	0.19
Treatment			
mPSL pulse therapy	7 (43.8)	6 (75.0)	0.21
CS + CNI	14 (87.5)	3 (28.6)	0.02
CS + CNI + cyclophosphamide	2 (12.5)	5 (71.4)	
Laboratory indices (at 1 month after treatment)			
Ferritin, ng/mL	344 [161–671]	539 [293–1582]	0.77
KL-6, U/mL	841 [680–946]	1415 [1064–1575]	0.09
SP-D, ng/mL	42.6 [29.9–88.3]	123.6 [42.8–176.0]	0.19
IFN-λ3, pg/mL	12.7 [6.3–22.0]	118.8 [72.2–172.0]	0.004

Data are presented as the median [interquartile range] or number (%).

CADM, Clinically amyopathic dermatomyositis; CK, Creatine kinase; CRP, C-reactive protein; CS, Corticosteroids; CNI, Calcineurin inhibitors; DM, Dermatomyositis; FEV_1_, Forced expiratory volume in 1 s; FVC, Forced vital capacity; IFN-λ3, Interferon-lambda 3; ILD, Interstitial lung disease; KL-6, Krebs von den Lungen-6; MDA5, Melanoma differentiation-associated gene 5; mPSL, Methylprednisolone; SP-D, Surfactant protein-D; WBC, White blood cell.

Regarding ILD subtype, the good control group had a higher proportion of chronic ILD, whereas the poor control group had more cases of the acute form, although this difference was not statistically significant. Notably, no significant differences in respiratory function tests or laboratory data at diagnosis were observed between the groups. Additionally, the poor control group was more frequently treated with triple therapy (corticosteroid, tacrolimus and intravenous cyclophosphamide) compared to the good control group. Serum IFN-λ3 levels at 1 month after treatment were significantly higher in the poor control group than in the good control group (*p* = 0.004). Serum KL-6 levels tended to be higher in the poor control group; however, the difference was not statistically significant.

Furthermore, when the analysis was restricted to mortality alone, serum IFN-λ3 levels at 1 month after treatment initiation remained significantly higher in patients who died (*n* = 6) than those in survivors (*n* = 18) (median 90.3 vs. 15.4 pg/mL, *p* = 0.049). In addition, overall survival stratified by serum IFN-λ3 levels at 1 month after treatment initiation is presented in Supplementary Fig. S1. Although statistical significance was not reached when patients were divided into two groups according to the median serum IFN-λ3 level (21.4 pg/mL), a trend toward poorer overall survival was observed in patients with higher IFN-λ3 levels compared with those with lower IFN-λ3 levels (log-rank *p* = 0.0502).

### Serum IFN-λ3 levels reflect disease control in anti-MDA5 antibody-positive DM-ILD

**Fig. 2:**
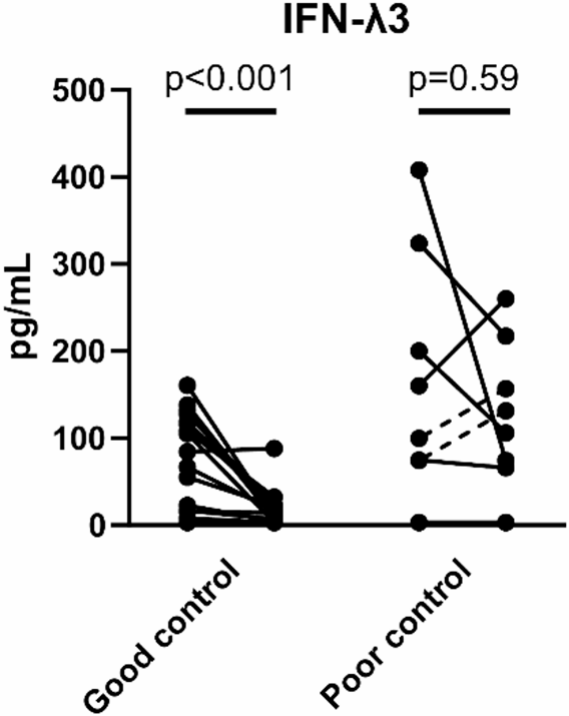
Changes in serum IFN-λ3 levels before and after treatment in anti-MDA5 antibody-positive DM-ILD.

We next compared sequential changes in serum IFN-λ3 levels from diagnosis to 1 month after treatment initiation between the good and poor control groups. As shown in Fig. 2, individual patients demonstrated varied changes in serum IFN-λ3 levels.

We analyzed clinical parameters in two groups: the good control group and the poor control group. Serum IFN-λ3 levels were measured at baseline and 1 month after treatment initiation. Dotted lines in the poor control group indicate patients who experienced relapse but survived.

DM, dermatomyositis; IFN-λ3, interferon-lambda 3; ILD, interstitial lung disease; MDA5, melanoma differentiation-associated gene 5.

In the good control group, serum IFN-λ3 levels significantly decreased from 94.6 pg/mL at diagnosis to 12.7 pg/mL at 1 month (*p* < 0.001). In contrast, the poor control group showed no significant reduction, with levels changing from 129.9 pg/mL at diagnosis to 118.8 pg/mL at 1 month after treatment (*p* = 0.59). Although four patients in the poor control group showed a decreasing trend in serum IFN-λ3 levels from baseline, their absolute IFN-λ3 levels at 1 month remained high, with the lowest value being 65.9 pg/mL. These findings suggest that persistent elevated IFN-λ3 levels at 1 month may reflect insufficient disease control and the risk of progression or relapse in anti-MDA5 antibody-positive DM-ILD.

**Fig. 3:**
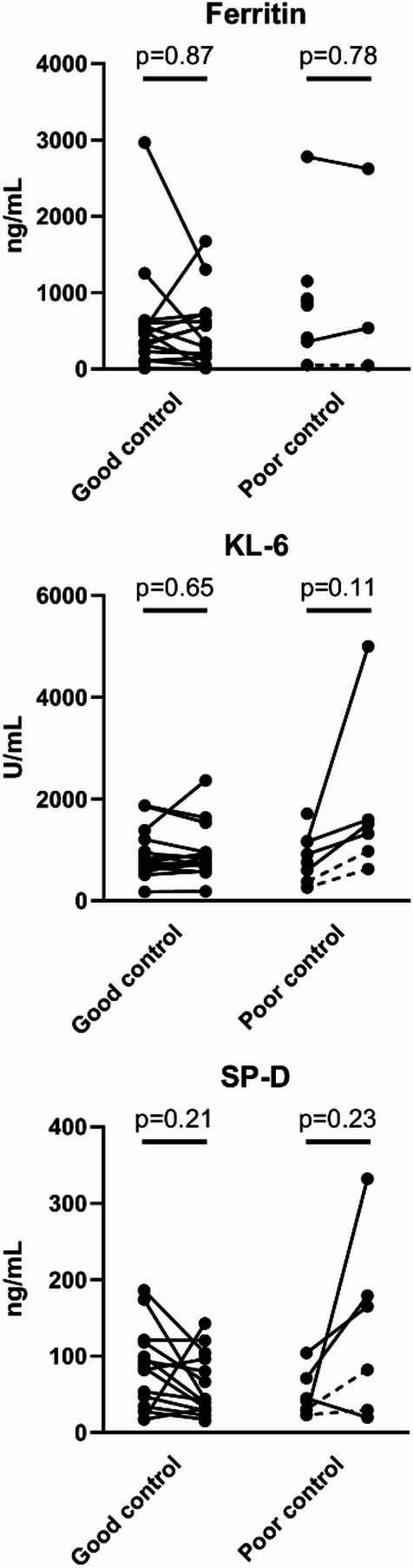
Changes in laboratory indices before and after treatment in patients with anti-MDA5 antibody-positive DM-ILD.

We also evaluated changes in other serum biomarkers, including ferritin, KL-6 and SP-D, between diagnosis and 1 month after treatment (Fig. 3).

Laboratory parameters were measured at baseline and 1 month after treatment initiation. Patients were categorized into two subgroups: good control group and poor control group. Dotted lines in the poor control group indicate patients who experienced relapse but survived.

DM, dermatomyositis; ILD, interstitial lung disease; KL-6, Krebs von den Lungen-6; MDA5, melanoma differentiation-associated gene 5; SP-D, surfactant protein-D.

None of these markers showed significant changes over this period in either group, indicating that ferritin, KL-6 and SP-D may not be reliable biomarkers for short-term disease control or treatment response. Additionally, serum levels of ferritin, KL-6 and SP-D at 1 month did not differ significantly between the two groups (Table 2).

## Discussion

This is the first study to evaluate short-term changes in serum IFN-λ3 levels in patients with anti-MDA5 antibody-positive DM-ILD. We found that serum IFN-λ3 levels varied considerably during the early clinical course and reflected both treatment response and ILD disease control. Specifically, the good control group showed a significant reduction in serum IFN-λ3 levels after initial treatment, whereas no such reduction was observed in the poor control group. Furthermore, serum IFN-λ3 levels at 1 month after treatment initiation were significantly lower in the good control group than in the poor control group. These findings suggest that serum IFN-λ3 may serve as a useful sequential biomarker for assessing disease control and short-term treatment response in patients with anti-MDA5 antibody-positive DM-ILD.

A key finding of this study is that persistently elevated absolute serum IFN-λ3 levels at 1 month after treatment initiation were closely associated with poor disease control of ILD. In our previous study, we have shown that high serum IFN-λ3 levels at diagnosis were significantly associated with poor prognosis in patients with anti-MDA5 antibody-positive DM-ILD^14^. However, no data were available on how these levels change over the clinical course in relation to treatment response. To address this gap, we evaluated serial serum IFN-λ3 levels at diagnosis and 1 month after treatment initiation. Although short-term reductions in IFN-λ3 levels were observed in some patients regardless of outcome, patients in the poor control group—those who either died from ILD progression or experienced ILD relapse within 1 year—generally retained high absolute IFN-λ3 levels at 1 month. In contrast, patients in the good control group—those who improved with initial therapy and maintained a stable condition—consistently achieved low absolute IFN-λ3 levels at this time point. These findings indicate that failure to achieve low absolute serum IFN-λ3 levels at 1 month after treatment initiation, even in the presence of a short-term decreasing trend, may predict subsequent mortality or ILD relapse in patients with anti-MDA5 antibody-positive DM-ILD. Thus, absolute IFN-λ3 levels at 1 month may be more clinically informative than relative changes from baseline.

In this study, we assessed serum KL-6 and ferritin levels in addition to serum IFN-λ3 levels, as all these biomarkers are known to be associated with poor outcomes in patients with anti-MDA5 antibody-positive DM-ILD^5–7^. Regarding KL-6 and ferritin, no significant changes were observed between diagnosis and 1 month after treatment initiation. Previous studies have reported that KL-6^5^ and ferritin^7^ are useful for monitoring disease control in patients with rapidly progressive anti-MDA5 antibody-positive ILD. However, these markers typically fluctuate over a longer clinical course, often more than 2 months after treatment initiation.

In contrast, our study demonstrated that serum IFN-λ3 levels declined rapidly within 1 month in patients who responded well to treatment and remained clinically stable (the good control group). Given that anti-MDA5 antibody positivity is associated with a high 90-day mortality rate^1,27^, and that pulmonary involvement is the principal determinant of short-term deterioration and mortality in anti-MDA5 antibody-positive DM-ILD^2,3^, timely assessment of ILD treatment response during the early clinical course is crucial for guiding therapeutic decision-making. Our findings suggest that serum IFN-λ3 may serve as a valuable early-phase biomarker for evaluating initial treatment response and short-term disease control in patients with anti-MDA5 antibody-positive DM-ILD.

Previous studies have demonstrated associations between serum IFN-λ3 levels and disease activity or severity across a range of autoimmune and inflammatory conditions. In SLE, elevated serum IFN-λ3 levels have been observed in patients with high disease activity and hypocomplementemia^28^, while in SSc, higher IFN-λ3 levels have been reported in patients with pulmonary fibrosis^12^, suggesting a link with fibrotic organ involvement. In addition, serum IFN-λ3 has been shown to reflect disease severity in Coronavirus disease 2019, increasing prior to the development of severe disease and declining rapidly during recovery^21^, consistent with a transient immune response during the short-term clinical course. In line with these observations, our study found that serial changes in serum IFN-λ3 levels were associated with disease control in patients with anti-MDA5 antibody-positive DM-ILD.

The pathogenic mechanisms underlying anti-MDA5 antibody–positive DM-ILD remain unclear. Viral infections have been proposed as potential triggers, as MDA5 is a key RNA sensor in the retinoic acid-inducible gene I-like receptor family that recognizes double-stranded RNA viruses^29^. MDA5 plays a central role in antiviral immunity by inducing type I interferon production in response to viral infection. Consistent with this, recent studies have reported upregulation of type I interferon signalling in the skin of patients with anti-MDA5 antibody-positive DM, suggesting a contribution to cutaneous damage^30–32^. Against this background, the role of IFN-λ3 in the pathogenesis of anti-MDA5 antibody-positive DM-ILD remains incompletely understood. Type III interferons, including IFN-λ3, signal through the JAK–STAT pathway and are produced in response to pathogen-associated molecular patterns, similar to type I interferons. In our previous lung histopathological analysis, IFN-λ3-positive staining was observed in macrophages, airway epithelial cells, the pleura, and intrapulmonary veins in patients with anti-MDA5 antibody-positive DM-ILD^14^. Although increased IFN-λ3 expression and activation of IFN-λ signalling pathways may contribute to disease pathogenesis, further studies are required to clarify its precise role in disease progression.

Several limitations of this study should be acknowledged. First, as a retrospective study, it was difficult to standardize treatment protocols and the timing of serum sample collection across patients. To minimize variability, we restricted the study population to patients with available serum samples collected 1 month after treatment initiation. Second, the study cohort was relatively small, reflecting the rarity of anti-MDA5 antibody-positive DM-ILD. Third, patients who died within the first month after treatment initiation were necessarily excluded because follow-up serum samples were unavailable, which may have introduced selection bias toward patients who survived the early fulminant phase. Fourth, we could not determine whether differences in treatment regimens influenced serum IFN-λ3 levels. Although the poor control group received more intensive therapy (e.g., corticosteroids, tacrolimus, and intravenous cyclophosphamide) than the good control group, they still exhibited higher serum IFN-λ3 levels at 1 month after treatment, suggesting that IFN-λ3 may reflect disease control and treatment response independently of treatment intensity. Prospective multicenter studies with larger cohorts and serial longitudinal measurements of serum IFN-λ3 are warranted to confirm the reproducibility of these findings.

In conclusion, we evaluated the trends in serum IFN-λ3 levels during the short-term clinical course in patients with anti-MDA5 antibody-positive DM-ILD. Our results demonstrated that patients who responded to initial treatment and remained clinically stable exhibited a significant reduction in serum IFN-λ3 levels after treatment initiation. In contrast, persistently elevated serum IFN-λ3 levels were associated with poor disease control, including increased mortality and ILD relapse rates. These findings indicate that serum IFN-λ3 levels may serve as a useful sequential biomarker for evaluating disease control, particularly in the early stages of treatment, in patients with anti-MDA5 antibody-positive DM-ILD.

## Supplementary Information

Below is the link to the electronic supplementary material.

Supplementary Material 1

## Data Availability

The data that support the findings of this study are available from the corresponding authors upon reasonable request.
