# Supplementary material for: Serum interferon-λ3 as a short-term biomarker of disease control in anti-MDA5-positive dermatomyositis-associated ILD
Source: Sci Rep. 2026 Jan 24;16:6134. doi: 10.1038/s41598-026-37104-x (PMC12901154; doi:10.1038/s41598-026-37104-x)
Supplement: Supplementary file 1 — Supplementary Material 1 [file 41598_2026_37104_MOESM1_ESM.docx]

**Supplementary Information**

**Supplementary Table S1**. Details of relapse cases within 1 year in anti-MDA5 antibody–positive DM-ILD

| Patient | Age (years) | Sex | Form of ILD | Time to relapse (months) | Relapse of ILD | Relapse of skin manifestation | Baseline  CNI | PSL dose before relapse (mg/day) | Intensified treatment at relapse | | Response to intensified treatment | Outcome |
| --- | --- | --- | --- | --- | --- | --- | --- | --- | --- | --- | --- | --- |
|  |  |  |  |  |  |  |  |  | Adjusted PSL dose (mg/day) | Additional Immunosuppressive agents |  |  |
| 1 | 32 | Female | Chronic | 5.9 | + | + | Tacrolimus | 15 | 15 | IVCY | + | Survived |
| 2 | 50 | Female | Subacute | 3.6 | + | − | Tacrolimus | 20 | 50 | IVCY | + | Survived |

Abbreviations: CNI, calcineurin inhibitor; DM, dermatomyositis; DM-ILD, dermatomyositis-associated interstitial lung disease; IFN-λ3, interferon-lambda 3; ILCY, intravenous cyclophosphamide; ILD, interstitial lung disease; MDA5, melanoma differentiation-associated gene 5; PSL, prednisolone.

**Supplementary Table S2**. Baseline characteristics of patients with anti-MDA5 antibody–positive DM-associated ILD, stratified by availability of early follow-up serum samples

|  | Patients with early follow-up serum | Patients without early follow-up serum |  |
| --- | --- | --- | --- |
|  | (n = 24) | (n = 31) | P value |
| **Age**, years | 53 [47–65] | 56 [46–67] | 0.36 |
| **Sex**, male | 7 (29.2) | 5 (16.1) | 0.33 |
| **Diagnosis** |  |  |  |
| – DM | 9 (37.5) | 17 (54.8) | 0.28 |
| – CADM | 15 (62.5) | 14 (45.2) |  |
| **Form of ILD** |  |  |  |
| – Acute | 7 (29.2) | 12 (38.7) | 0.53 |
| – Subacute | 10 (41.7) | 14 (45.2) |  |
| – Chronic | 7 (29.2) | 5 (16.1) |  |
| **Smoking status**, former | 11 (45.8) | 8 (25.8) | 0.16 |
| **Symptoms** |  |  |  |
| – Fever | 10 (41.6) | 15 (48.4) | 0.79 |
| – Cough | 14 (58.3) | 18 (58.1) | 1 |
| – Dyspnea | 16 (66.7) | 21 (67.7) | 1 |
| – Fine crackles | 21 (87.5) | 25 (80.6) | 0.72 |
| – Arthralgia | 12 (50.0) | 16 (51.6) | 1 |
| – Gottron’s papule | 21 (87.5) | 31 (100.0) | 0.77 |
| – Heliotrope rash | 9 (37.5) | 14 (45.2) | 0.60 |
| – Skin ulcer | 4 (16.7) | 8 (25.8) | 0.52 |
| – Muscle weakness | 9 (37.5) | 15 (48.4) | 0.58 |
| – Muscle pain | 8 (33.3) | 8 (25.8) | 0.57 |
| **Pulmonary function indices** (n = 47) |  |  |  |
| – FVC, L | 2.2 [1.7–2.7] | 2.1 [1.8–2.4] | 0.69 |
| – %FVC | 75.7 [68.0–80.5] | 76.4 [54.3–94.7] | 0.67 |
| – FEV_1_, L | 1.7 [1.5–2.7] | 1.8 [1.4–2.0] | 0.92 |
| – %FEV_1_ | 68.4 [57.0–84.0] | 74.0 [58.9–93.9] | 0.47 |
| **Pulmonary diffusion capacity** (n = 38) |  |  |  |
| – DLco, mL/min/mmHg | 13.2 [10.5–15.5] | 12.3 [9.3–14.1] | 0.58 |
| – %DLco | 63.1 [54.6–85.3] | 66.0 [50.9–85.3] | 0.85 |
| **PaO₂**, Torr (n = 51) | 77.5 [64.8–84.5] | 73.5 [68.0–85.2] | 0.77 |
| **P/F ratio** (n = 54) | 357 [292–401] | 350 [324–406] | 0.63 |
| **Serum IFN-λ3,** pg/mL | 102.4 [46.9–133.5] | 124.3 [69.3–195.0] | 0.16 |
| **Treatment** |  |  |  |
| – mPSL pulse therapy before maintenance therapy | 13 (54.2) | 23 (74.2) | 0.16 |
| – CS + CNI | 17 (70.8) | 22 (71.0) | 1 |
| – CS + CNI + cyclophosphamide | 7 (29.2) | 9 (29.0) |  |
| **Mortality** | 6 (25.0) | 7 (25.0) | 1 |
| **Relapse of ILD <1 year** | 2 (8.3) | 4 (12.9) | 0.69 |

Data are presented as the median [interquartile range] or number (%).

CADM, clinically amyopathic dermatomyositis; CS, corticosteroids; CNI, calcineurin inhibitors; DLco, diffusion capacity of the lung for carbon monoxide; DM, dermatomyositis; FEV_1_, forced expiratory volume in 1 second; FVC, forced vital capacity; IFN-λ3, interferon-lambda 3; ILD, interstitial lung disease; MDA5, melanoma differentiation-associated gene 5; mPSL, methylprednisolone; PaO₂, partial pressure of arterial oxygen; P/F ratio, PaO₂/fraction of inspired oxygen ratio.

**Supplementary Figure S1**. Association of serum IFN-λ3 levels at early follow-up with overall survival in anti-MDA5 antibody-positive DM-ILD.
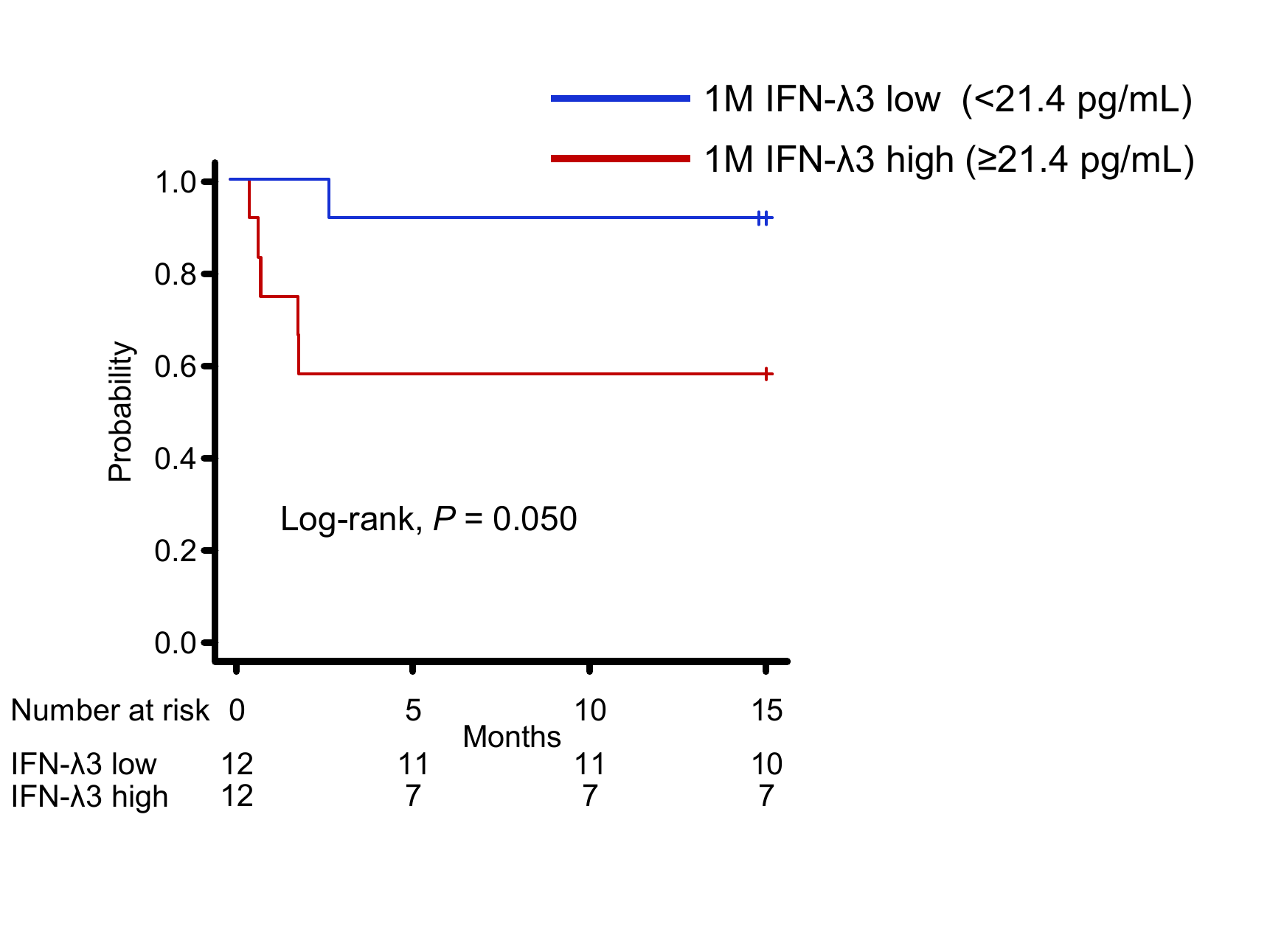


Kaplan-Meier curve shows the overall survival. Patients were stratified according to serum IFN-λ3 levels measured at one month after treatment initiation, using the median value (21.4 pg/mL) as the cutoff (low, <21.4 pg/mL vs high, ≥21.4 pg/mL). *P*-values were calculated using the log-rank test.

Abbreviations: DM, dermatomyositis; IFN-λ3, interferon-lambda 3; ILD, interstitial lung disease; MDA5, melanoma differentiation-associated gene 5.
